# Arthroscopic Rotator Cuff Repair and Subsequent Career Changes in Manual Workers: A Long-term Observational Study

**DOI:** 10.2106/JBJS.OA.25.00311

**Published:** 2026-01-16

**Authors:** Philippe Collin, Donald Tedah, Leonard Stokes, Patrick J. Denard, Angélique Delarue, Céline Daniel, Alexandre Lädermann

**Affiliations:** 1 Saint-Grégoire Private Hospital, Saint-Grégoire, France; 2 Clinic Victor Hugo, Paris, France; 3 American Hospital, Paris, France; 4 University of Brescia, Brescia, Italy; 5 Oregon Shoulder Institute, Medford, Oregon; 6 Division of Orthopaedics and Trauma Surgery, La Tour Hospital, Meyrin, Switzerland; 7 Faculty of Medicine, University of Geneva, Genève, Switzerland; 8 Division of Orthopaedics and Trauma Surgery, Department of Surgery, Geneva University Hospitals, Geneva, Switzerland; 9 FORE (Foundation for Research and Teaching in Orthopedics, Sports Medicine, Trauma, and Imaging in the Musculoskeletal System), Meyrin, Switzerland

## Abstract

**Background:**

Although arthroscopic rotator cuff repair (ARCR) is widely performed for rotator cuff tears, the extent to which manual laborers can return to their original occupations remains unclear. This study aimed to evaluate the proportion of manual workers who change or discontinue their professional activity following ARCR.

**Methods:**

A retrospective review was conducted of patients who underwent ARCR by a single surgeon between December 2010 and June 2014. Inclusion criteria were a minimum follow-up of 9 years and employment in a manual labor occupation before surgery. Patients completed a long-term follow-up assessment, which included a professional activity questionnaire (primary outcome) and shoulder function evaluations (secondary outcome).

**Results:**

A total of 165 patients (55 women, 110 men; mean age 53 ± 5 years at the time of surgery) were followed for an average of 11 ± 1 years. Of these, 128 patients (77%) resumed the same occupation postoperatively, while 37 patients (23%) changed or discontinued their work. There was no group difference for the subjective shoulder value at the final follow-up between physical workers and repetitive workers (p = 0.75). A significant association was found between occupational change and poorer shoulder function, as reflected by lower Constant scores (p = 0.008). The median time to occupational change was 12 months (interquartile range: 6-60 months). The mean was 29.7 ± 33.8 months. Change in professional activity was not associated with the presence of a full-thickness retear (p = 0.70), initial tear type (p = 0.23), or sex (p = 0.09). However, salaried employees were more likely to change their occupation than self-employed individuals (p = 0.02).

**Conclusions:**

Most manual workers can maintain their original occupation following ARCR. However, approximately 1 in 4 patients changes or abandons their job, potentially due to suboptimal shoulder function. Self-employed individuals appear less likely to modify their professional activity.

**Clinical Relevance:**

Manual laborers undergoing ARCR can generally expect to resume their professional duties. Nevertheless, persistent shoulder symptoms may necessitate a career transition in some cases.

**Level of Evidence:**

Level IV. See Instructions for Authors for a complete description of levels of evidence.

## Introduction

Return to work after arthroscopic rotator cuff repair (ARCR), particularly in physically demanding jobs, remains a major concern for patients and clinicians^1^. Although clinical outcomes are well documented, occupational recovery is less predictable and influenced by nonsurgical factors.

Reported return to work rates usually exceed 60%, but only about 40% of patients resume their preinjury work level^1-6^. Manual laborers are less likely to return to full duties, even with satisfactory clinical and structural results^7^. Many experience persistent limitations that impede full reintegration. Most studies assess short-term to mid-term follow-up (6-12 months)^1^, which may underestimate delayed recovery or later workforce withdrawal due to residual symptoms, reinjury, or job constraints^8^. No study has yet evaluated long-term return to work after ARCR, leaving a gap in understanding its sustained vocational impact.

This study aimed to determine the proportion of manual workers who change occupation at long-term follow-up after ARCR. We hypothesized that those with retears would be less likely to resume their occupation than those with successful repairs. Secondary outcomes were predefined and included long-term shoulder function assessed by the Constant score and subjective shoulder value (SSV), the time to occupational change, and potential associations with work-related variables such as job type and employment status.

## Methods

### Study Design and Patient Selection

Ethical approval was obtained from our Institutional Review Board (CERC-VS-2023-07-3). All patients provided informed consent.

A retrospective single-center, observational cohort study was performed of ARCRs performed by a single surgeon between December 2010 and June 2014. Patients were regularly followed up by the senior author (P.C.) systematically at 6 weeks, 3 months, and 6 months postoperatively. Eligible patients had undergone ARCR at the age of 18 years or older, were manual laborers at the time of surgery, and had a minimum follow-up of 9 years. Patients were excluded if they (1) held occupations classified as light-duty work (e.g., data entry or office-based jobs), (2) underwent revision surgery on the same shoulder, or (3) had comorbid conditions that could confound shoulder function, such as advanced glenohumeral arthritis or prior proximal humerus fractures. Patients with occupational disease or work-related injury (workers' compensation) were also included (8% and 15% of the cohort, respectively). No subgroup analysis was performed for these categories, as some cases were still pending official recognition, and the study focused instead on job type (heavy vs. repetitive) and employment status (salaried vs. self-employed). Additional exclusion criteria included incomplete clinical records or lack of standard postoperative follow-up. Eligible patients were contacted by telephone and were asked to participate in the study. Those who agreed were invited to return for a last follow-up assessment. Of note, the patients in this study were not asked about their race or ethnicity, so this information was not included in the study.

### Study Variables

The main outcome was whether patients had changed occupation since surgery, determined through a follow-up phone call. Factors associated with a change in professional activity were also identified.

Baseline characteristics included age, sex, weight, height, shoulder side, limb dominance, and rotator cuff lesion features (size, location, etiology). Preoperative work-related variables were collected via standardized forms: recognition as an occupational disease, job nature (physically demanding or repetitive), and employment status (salaried or self-employed). These parameters were analyzed for associations with postoperative occupational outcomes.

### Outcome Measures

The primary outcome was assessed through a one-time follow-up call regarding professional activity before and after ARCR, including whether any change was linked to shoulder symptoms.

The secondary outcome was early postoperative rotator cuff healing, assessed at the 6-month follow-up using the Sugaya classification, which ranges from I (no indication of retear) to V (full-thickness retear)^9^, and determined by ultrasound (US)^10^. This assessment was performed by a trained musculoskeletal radiologist.

Nonmassive tears involved the supraspinatus or superior/entire subscapularis. Massive tears were classified per Collin^11^: type A (supraspinatus + superior subscapularis), B (supraspinatus + entire subscapularis), C (supraspinatus + superior subscapularis + infraspinatus), D (supraspinatus + infraspinatus), and E (supraspinatus + infraspinatus + teres minor).

Secondary functional outcomes included 2 shoulder scores. The SSV rates shoulder function from 0% (unusable) to 100% (normal)^12,13^. The Constant score^14^ assesses pain (/15), daily activities (/20), mobility (/40), and strength (/25), with higher scores indicating better function. Constant scores were available preoperatively and postoperatively.

### Statistical Analyses

The sample size was based on convenience sampling of eligible patients. Quantitative data were summarized as means, standard deviations, medians, and interquartile ranges (IQR) and compared using *t*-tests. Qualitative data were expressed as frequencies and percentages and analyzed using χ^2^ tests. No imputation was performed for missing data.

## Results

Of 200 potentially eligible patients, 35 were excluded: 26 unreachable, 3 refused, 3 deceased, 1 with Alzheimer disease, 1 treated elsewhere, and 1 with shoulder replacement. Follow-up data were available for 165 patients (mean age 53 ± 5 years at surgery).

**TABLE I:** Demographic and Clinical Characteristics of the Study Participants

	Total (n = 165)
Sex (N, %)	
Female	55 (33)
Male	110 (67)
Age, years	
Mean (SD)	53 (5)
Range	26-68
Weight, kg (mean, SD)	76 (15)
Height, cm (mean, SD)	168 (9)
Handedness (N, %)	
Right	156 (94)
Left	8 (5)
Ambidextrous	1 (1)
Tear type (N, %)	
Isolated supraspinatus	88 (53)
Isolated superior subscapularis	10 (6)
Entire subscapularis	8 (5)
Type A	15 (9)
Type B	7 (4)
Type C	9 (6)
Type D	25 (15)
Type E	3 (2)
Side of tear (N, %)	
Right	116 (70)
Left	49 (30)
Cause of tear (N, %)	
Accident at work	24 (15)
Occupational disease	13 (8)
Not known	128 (77)
Job type (N, %)	
Physical (e.g., builder, farmer)	149 (90)
Repetitive (e.g., cashier, factory work)	16 (10)
Employment type (N, %)	
Salaried	100 (60)
Self-employed	65 (40)

Data are shown for the time of surgery. The types of rotator cuff tear are based on a previous article^11^.

The last follow-up was 11 ± 1 years after surgery. Two-thirds of the patients were men (110; 67%), and a third were women (55; 33%; sex assigned at birth). Most of the patients had jobs involving heavy physical work at the time of surgery (149; 90%), with just 16 (10%) employed in repetitive work (Table I). The exact occupations varied considerably, but many patients were farmers (40 patients) or carpenters/joiners (20 patients).

At the final follow-up, patients’ mean age was 64 ± 5 years. Most (105; 63.7%) were retired, while 49 (29.7%) remained employed.

### Primary Outcome

At the last follow-up, 37 patients (23%) reported changing or leaving their job after surgery (Table II): 14 (38%) remained off work until retirement or on disability and 23 (62%) switched occupations. Among them, 68% (25/37) cited shoulder symptoms as the reason, 22% not, and 10% were unspecified. Eleven patients reported timing of this change, ranging from immediately post-surgery to 10 years later (median 24 months, IQR 6-60). Reported new occupations included truck driving, factory work, and gardening.

**TABLE II:** Professional Activity Following ARCR

	No. of Patients (Total: 165)
Changed professional activity (N, %)	
Yes	37 (23)
No	128 (77)
Current activity (%)	
Retired	105 (63.7)
Working	49 (29.7)
Disabled	5 (3)
Sick leave for other shoulder	1 (0.6)
Sick leave for other reason	4 (2.4)
On leave for occupational disease	1 (0.6)

ARCR = arthroscopic rotator cuff repair.

Data are shown for the last follow-up.

### Secondary Outcome

**Fig. 1:**
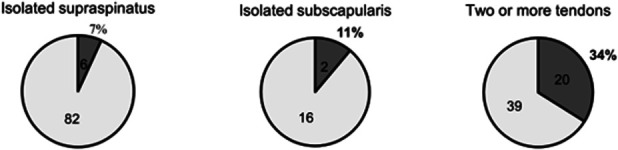
Full-thickness retears following arthroscopic rotator cuff repair according to the original tear type. (The number of patients with full-thickness retears (Sugaya classification IV-V) is shown in the dark gray segment. The number of patients who did not have full-thickness retears is shown in the pale gray segment.)

There were 28 patients (17%; 28/165) who had full-thickness retears (Sugaya classification IV–V), as identified on the routine 6-month follow-up ultrasound. Retears varied based on the original tear pattern (p < 0.0001), with a higher incidence of full-thickness retears when the original tear involved 2 or more tendons (34% vs. 7% and 11%; Fig. 1). There was no difference in retear rate among those that did not change their occupation (18%; 23/128) compared with those who changed their activity (14%; 5/37) (p = 0.70).

### Shoulder Function Scores

There was no group difference for the SSV at final follow-up between groups (p = 0.75). The mean SSV score was 80 ± 13 at the last follow-up.

**Fig. 2:**
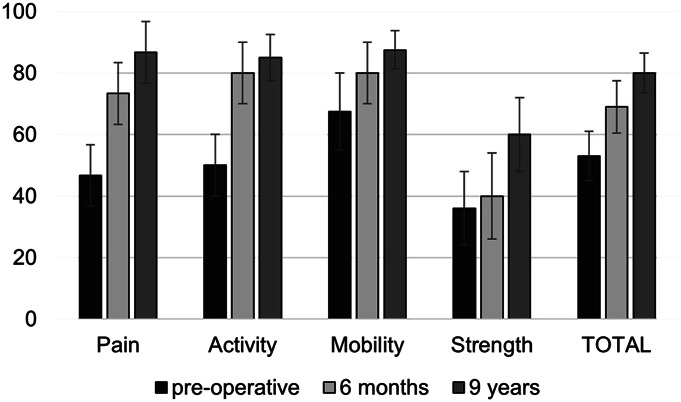
Constant scores before arthroscopic rotator cuff repair and after 6 months and 9 or more years. (The mean scores are shown with error bars representing the standard deviation. Note that the subscores have all been converted to percentage scores. Higher scores indicate better shoulder function.)

Constant scores improved across all subscales over time (Fig. 2). At the last follow-up, the mean Constant score was 80 ± 13. Patients who changed occupation had significantly lower Constant scores at 6 months (62 vs. 71; p = 0.02) and at the final follow-up (73 vs. 82; p = 0.008), but not preoperatively (55 vs. 52; p = 0.51). SSV was also lower in this group at the last follow-up (74 vs. 82; p = 0.009). No Constant score differences were observed between heavy physical and repetitive workers before surgery (53 vs. 57; p = 0.71) or at the last follow-up (80 vs. 74; p = 0.12). However, at 6 months, heavy workers showed higher scores (70 vs. 54; p = 0.007).

### Additional Factors Affecting the Change in Professional Activity

Among those doing repetitive work before surgery 37.5% (6/16) changed occupations, while 20.8% (31/149) of those doing heavy physical work before surgery changed occupations (p = 0.23). There was no difference in rotator cuff tear pattern and change in occupation (p = 0.23) (Table III).

**TABLE III:** Postoperative Professional Activity According to the Type of ARCR

ARCR	Changed Professional Activity (%)	Did Not Change Professional Activity (%)	Total
Isolated supraspinatus	23 (26)	65 (74)	88
Isolated superior subscapularis	4 (40)	6 (60)	10
Entire subscapularis	3 (38)	5 (62)	8
Type A	1 (7)	14 (93)	15
Type B	1 (14)	6 (86)	7
Type C	0 (0)	9 (100)	9
Type D	4 (16)	21 (84)	25
Type E	1 (33)	2 (67)	3

ARCR = arthroscopic rotator cuff repair.

Salaried workers were more likely to change their profession following surgery compared with self-employed workers [29% (29/100) vs. 12% (8/65); p = 0.02]. There were no sex-based differences in the rate of profession change [women: 31% (17/55) vs. men: 18% (20/110); p = 0.09].

## Discussion

This study found that 77% of manual workers maintained their occupation after ARCR, indicating that most patients can resume physically demanding jobs, contrary to our initial hypothesis. Among the 23% who changed occupations, most cited persistent shoulder symptoms, and these patients had significantly lower functional scores, highlighting the effect of residual dysfunction on work capacity

Our results align with previous studies reporting high return-to-work rates after ARCR. Two recent reviews found rates of 62% and 83.8%^2,4^. Similarly, one 12-month study reported that 67% of patients remained in the same job.^3^ Variations likely reflect differences in the proportion of manual laborers and tear severity.^1^

We investigated factors associated with occupational change. Although heavy physical work has been linked to poorer return to work outcomes^1,3,15^, we found no association. This may reflect the predominance of manual workers (90%) in our cohort. Salaried workers were more likely to change occupation than self-employed individuals, possibly because self-employed workers can adjust their workload or due to job type differences. Tear pattern was not significantly related to occupational change.

Previous studies have suggested higher rates of occupational change among women^3,15^. Our data showed a similar trend (31% vs. 18% for men) without statistical significance, possibly due to sample size. Conversely, one study found that women more likely to return to work, attributed to less strenuous jobs^16^.

Our study found that less than one-fifth of the patients had full-thickness retears. This is close to the rates reported in a recent review^17^, although previous studies have shown considerable variation due to factors such as the size of the tear and the patients' age^17-19^. We found that full-thickness retears mainly affected patients who had initial tears involving 2 or more tendons. Notably, retears were not associated with changes in professional activity. This suggests that structural healing alone may not be the main determinant of occupational outcomes. Other factors that were not assessed in our study such as job satisfaction, the presence of Workers’ Compensation claims, or medical comorbidity burden (e.g., Charlson Comorbidity Index) may have influenced whether patients continued or changed their occupation. These elements should be explored in future studies. This result can be linked to previous studies which found that ARCR improves functioning irrespective of whether the tendons heal or not^18,20^, although it should be noted that deterioration has been observed over the long-term when there are retears^20^.

It is relevant to note that the patients in our study often changed their professional activity after a delay of several years. It is possible that patients may have hoped for improvement over time and so initially persevered in their careers but then changed their occupation when this did not occur. Although we found postoperative improvements in shoulder function at 6 months which increased by 9 or more years, previous studies indicate that there is a plateau of maximum recovery at 1 year^21^. This could therefore potentially account for the median delay of 2 years to change activity. In terms of the speed of recovery, we found that the shoulder function at 6 months was better for patients who carried out heavy physical as opposed to repetitive work. This therefore suggests a potentially faster recovery in these patients possibly due to their physical fitness and everyday activities.

Several limitations of this study should be acknowledged. First, several factors which could influence occupational outcomes, such as job satisfaction, the presence of Workers’ Compensation claims, and medical comorbidity burden (e.g., Charlson Comorbidity Index), were not assessed. Second, although structural healing was generally successful, retears were not associated with changes in professional activity, suggesting that other unmeasured factors may have played a role. Third, the relatively small sample size limited subgroup analyses, particularly for women and those performing repetitive work. Fourth, the older age of some patients may have influenced retirement decisions independently of shoulder status. Finally, this was a single-center study with a male-predominant cohort, which may limit generalizability.

Overall, our study indicates that manual workers who undergo ARCR are likely to be able to continue their careers, but that a change in occupation could be considered if there are residual symptoms.

## Conclusion

Most manual laborers retain their occupation long-term following ARCR. However, 1 in 4 changes or leaves their profession, often due to residual symptoms. Self-employed individuals appear more likely to remain in their roles.
